# Tuberculosis‐Associated Renal Amyloidosis Presenting as Nephrotic Syndrome: A Case Report From Ethiopia

**DOI:** 10.1155/crin/3992146

**Published:** 2026-09-18

**Authors:** Alemayehu Abebe Guji, Daniel Rebuma Bekele, Getnet Amberber Degefu, Birhanu Gudeta Geleta, Derebe Shashigo Leilago, Kedir Negesso Tukeni

**Affiliations:** ^1^ Jimma University, Jimma, Ethiopia, ju.edu.et; ^2^ Saint Paul Hospital Millennium Medical College, Addis Ababa, Ethiopia

**Keywords:** Ethiopia, immunosuppression, Jimma, nephrotic syndrome, renal amyloidosis, tuberculosis

## Abstract

**Background:**

Tuberculosis (TB) is highly prevalent in Ethiopia, and extrapulmonary manifestations pose significant diagnostic challenges. Among its rare complications, renal amyloidosis (AA type) can present with nephrotic syndrome and progressive renal dysfunction. In resource‐limited settings, the absence of routine TB screening and restricted timely access to renal biopsy often lead to empirical immunosuppression, which may worsen outcomes by reactivating latent infections.

**Case Presentation:**

We report a case of a 43‐year‐old Ethiopian male with a 5‐month history of generalized edema, fatigue, flank pain, and nephrotic‐range proteinuria (19.7 g/24 h). Initial evaluation suggested high‐risk membranous nephropathy, and empirical immunosuppression with prednisone and cyclophosphamide was initiated before biopsy confirmation due to a delay in the renal biopsy results. The patient subsequently developed axillary lymphadenopathy and “B” symptoms. Renal biopsy results obtained after initiation of chemotherapy revealed Congo red–positive deposits with green birefringence under polarized light, consistent with AA amyloidosis. At the same time, lymph node histology demonstrated necrotizing granulomatous inflammation, confirming extrapulmonary TB. Anti‐TB therapy (RHZE regimen) led to initial clinical improvement and stabilization of the renal function. However, 3 weeks later, the patient developed severe pulmonary sepsis and died of septic shock despite intensive care.

**Conclusion:**

This case underscores the importance of excluding infectious etiologies, particularly TB, before initiating immunosuppressive therapy in patients with nephrotic syndrome in endemic regions. Empirical immunosuppression without histopathological confirmation risks reactivating latent TB and delaying appropriate treatment. Routine TB screening with IGRA or TST, combined with timely renal biopsy, should be prioritized in resource‐limited settings to prevent misdiagnosis and improve patient outcomes.

## 1. Introduction

Tuberculosis (TB) continues to be a major public health challenge in Ethiopia and other sub‐Saharan African countries, with an estimated incidence of 132 per 100,000 population in 2023. Despite global progress, TB remains a leading cause of morbidity and mortality, particularly in resource‐limited settings where diagnostic and therapeutic options are constrained [1]. While pulmonary TB is the most common presentation, extrapulmonary TB (EPTB) accounts for up to 30% of cases in Ethiopia, affecting diverse organ systems, including lymph nodes, pleura, and genitourinary tract [2]. These atypical manifestations often complicate timely diagnosis and delay appropriate treatment.

Renal involvement in TB is typically insidious, presenting as sterile pyuria, hematuria, or, rarely, nephrotic syndrome due to secondary (AA) amyloidosis. This condition arises from chronic inflammation, where serum amyloid A protein deposits in renal tissue, leading to heavy proteinuria, hypoalbuminemia, and progressive renal dysfunction. In developing countries, TB remains a leading cause of renal AA amyloidosis, which carries a poor prognosis if not treated promptly [3–5].

The diagnosis of TB‐associated renal amyloidosis is challenging in low‐resource settings. The clinical features are often nonspecific, and laboratory or imaging findings may be inconclusive. Histopathological confirmation via renal biopsy remains the gold standard; however, access is limited. Screening for latent TB using interferon‐gamma release assays (IGRA) or tuberculin skin tests (TST) can aid early detection, yet these tools are not routinely available in endemic regions [3, 4, 6].

This case report illustrates the diagnostic pitfalls of nephrotic syndrome in a TB‐endemic setting, emphasizing the need to exclude infectious causes—particularly TB—before initiating immunosuppressive therapy. It highlights the importance of heightened clinical suspicion, comprehensive evaluation, and context‐appropriate management strategies to improve outcomes in resource‐limited environments.

## 2. Case Presentation

A 43‐year‐old Ethiopian male with no prior medical history or known TB exposure presented with a 5‐month history of progressive generalized edema, fatigue, decreased urine output, flank pain, and pleuritic chest pain. He also reported a mild intermittent cough with scant sputum production, initially attributed to renal disease. He had no history of chronic medical conditions. He had no history of signs or symptoms suggestive of leprosy, which is a common chronic infectious condition in this setting.

## 3. Initial Diagnosis and Approach

The initial diagnosis was nephrotic syndrome based on the patient’s history, physical examination, and laboratory workups. Given the suspicion of high‐risk membranous nephropathy based on the KDIGO definition (proteinuria of > 8 mg/dL and a rapid decrement in eGFR; Table 1), the patient was empirically started on prednisone (60 mg/day) and IV cyclophosphamide at a dose of 120 mg/day while awaiting biopsy results because the expected turnaround time was more than 3 weeks, since the biopsy sample was sent outside Ethiopia due to a lack of the necessary resources and expertise for an examination of renal biopsy. Before the initiation of immunosuppression, the patient was evaluated for causes of primary amyloidosis, and there were no signs or symptoms suggestive of primary amyloidosis, including echocardiography assessment, which was within the physiological limit. Common secondary causes of amyloidosis and infectious conditions of nephrotic syndrome were also assessed and were unremarkable, including workup for HIV, hepatitis B, hepatitis C, malaria, and syphilis; autoimmune markers for rheumatologic conditions, such as rheumatoid arthritis and systemic lupus erythematosus, were also nonrevealing; workup for inflammatory bowel disease, which included upper gastrointestinal endoscopy and ileocolonoscopy, was unremarkable. Unfortunately, after a week of immunosuppressive treatment, his clinical condition worsened, and he developed lymphadenopathy and B symptoms, prompting further evaluation for TB.

**TABLE 1:** Laboratory and clinical parameters of nephrotic syndrome with renal dysfunction.

Parameter	Patient value	Reference range
24‐h urine protein	19,778 mg/24 h	< 150 mg/24 h

Serum albumin	0.47, 0.64, 0.7 g/dL	3.5–5.0 g/dL

Hyperlipidemia	• LDL: 259.6 mg/dL • TG: 379.1 mg/dL	• LDL: < 100 mg/dL • TG: < 150 mg/dL

Acute kidney injury	2.96/93.9, 4.04/85, 3.67/85, 3.74/85	• Cr: 0.6–1.2 mg/dL (F), 0.7–1.3 mg/dL (M); • Urea: 7–20 mg/dL

*Note:* Proteinuria (19,778 mg/24 h): severely elevated, indicating significant kidney damage or nephrotic syndrome. Serum albumin (0.47 g/dL): severely low, consistent with severe hypoalbuminemia. Hyperlipidemia: elevated LDL and triglycerides, increasing cardiovascular risk. Acute kidney injury: elevated creatinine and urea levels, indicating impaired kidney function.

## 4. Laboratory Findings

The patient demonstrated nephrotic‐range proteinuria (19.7 g/24 h), severe hypoalbuminemia (0.47–0.7 g/dL), hyperlipidemia (LDL 260 mg/dL, TG 380 mg/dL), and acute kidney injury (creatinine 2.96–4.04 mg/dL, urea 85–93.9 mg/dL). Hematological analysis revealed mild anemia (Hb = 11.7 g/dL) and thrombocytosis (450,000/µL). These findings were consistent with those of nephrotic syndrome complicated by renal dysfunction and metabolic derangements (Table 1).

Further laboratory profiles demonstrated mild anemia with reduced hemoglobin and hematocrit, preserved white cell and platelet counts, normal electrolytes, and marked renal dysfunction, as evidenced by elevated creatinine and urea levels. Liver enzymes remained within normal limits, while the lipid profile revealed severe dyslipidemia with markedly elevated total cholesterol, triglycerides, and LDL cholesterol levels, alongside preserved HDL levels, consistent with nephrotic syndrome complicated by renal impairment and metabolic derangements (Table 2).

**TABLE 2:** Comprehensive hematologic, renal, electrolyte, hepatic, and lipid profile parameters (patient vs. reference ranges).

Parameter	Patient value	Reference range
Date	8/12/24	—
Hematology		
White blood cell count	6 K/µL	4.0–11.0 K/µL
Neutrophils (%)	59%	40%–70%
Lymphocytes (%)	28%	20%–40%
Hemoglobin	11.7 g/dL	12–16 g/dL (F), 13.5–17.5 g/dL (M)
Hematocrit	33.4%	36%–46% (F), 40%–50% (M)
Mean corpuscular volume (MCV)	81 fL	80–100 fL
Platelet count	450 K/µL	150–450 K/µL
Electrolytes and renal function		
Sodium (Na)	138 mmol/L	135–145 mmol/L
Potassium (K)	4.5 mmol/L	3.5–5.0 mmol/L
Chloride (Cl)	104 mmol/L	98–107 mmol/L
Creatinine (Cr)	2.96 mg/dL	0.6–1.2 mg/dL (F), 0.7–1.3 mg/dL (M)
Urea	93.9 mg/dL	7–20 mg/dL
Liver function tests		
Alkaline phosphatase	57 U/L	44–147 U/L
Aspartate aminotransferase (AST)	29 U/L	10–40 U/L
Alanine aminotransferase (ALT)	20 U/L	7–56 U/L
Lipid profile		
Total cholesterol	344 mg/dL	< 200 mg/dL (desirable)
Triglycerides (TG)	380 mg/dL	< 150 mg/dL (normal)
Low‐density lipoprotein (LDL)	260 mg/dL	< 100 mg/dL (optimal)
High‐density lipoprotein (HDL)	55 mg/dL	> 40 mg/dL (M), > 50 mg/dL (F)

## 5. Histopathology and Imaging

**FIGURE 1:**
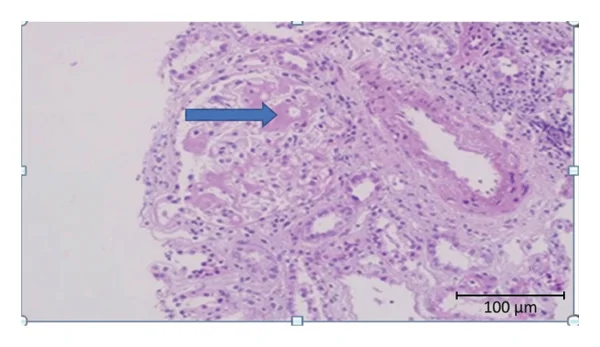
Renal biopsy (hematoxylin and eosin staining) showing diffuse eosinophilic mesangial matrix expansion due to amyloid deposition (blue arrows). The deposits exhibit the tinctorial properties of amyloid. Periodic acid–Schiff (PAS) and methenamine silver stains were negative, whereas Congo red staining showing characteristic green birefringence under polarized light, confirming the presence of amyloid.

Renal biopsy revealed diffuse irregular mesangial matrix expansion with eosinophilic deposits exhibiting amyloid characteristics on hematoxylin and eosin staining, confirmed by Congo red positivity and green birefringence under polarized light, while PAS and methenamine silver stains were negative. No tuft necrosis, endocapillary proliferation, subendothelial deposits, or thrombi were observed. Acute tubular injury was characterized by cytoplasmic vacuolization and occasional hyaline and granular casts, accompanied by mild (< 10%) cortical tubular atrophy, interstitial fibrosis, and focal chronic inflammation. The arteries and arterioles showed variably thickened walls with amyloid deposition, consistent with systemic AA amyloidosis involving the renal parenchyma and vasculature (Figure 1).

**FIGURE 2:**
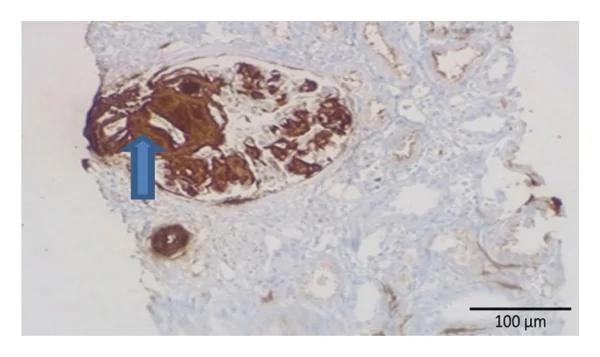
Immunohistochemical staining for serum amyloid A protein showing intense positivity along glomerular and extraglomerular sites of amyloid deposition (blue arrow). Immunofluorescence microscopy was negative for immunoglobulins (IgA, IgG, and IgM), complement components (C3 and C1q), and light chains (kappa and lambda), supporting AA amyloidosis.

Immunohistochemical staining for serum amyloid A protein demonstrated intense positivity along the glomerular and extraglomerular sites of amyloid deposition, confirming AA amyloidosis. Immunofluorescence microscopy (not shown) revealed negative findings for immunoglobulins (IgA, IgG, and IgM), complement components (C3 and C1q), and light chains (kappa and lambda), thereby excluding immune complex–mediated or light chain–associated pathologies (Figure 2).

**FIGURE 3:**
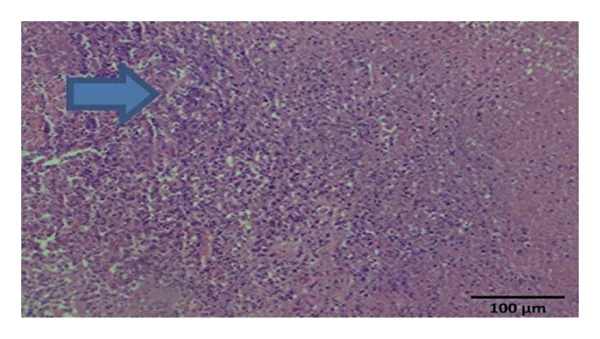
Excisional lymph node biopsy demonstrating necrotizing granulomatous inflammation with pinkish amorphous debris, scattered epithelioid cells, and ill‐formed granulomas (blue arrow), highly suggestive of tuberculous lymphadenitis.

Excisional lymph node biopsy revealed necrotic tissue with amorphous granular debris, scattered epithelioid cells, and ill‐formed granulomas, consistent with necrotizing granulomatous inflammation. These findings are highly suggestive of tuberculous lymphadenitis, characterized by caseous necrosis and poorly formed granulomas with epithelioid histiocytes (Figure 3).

**FIGURE 4:**
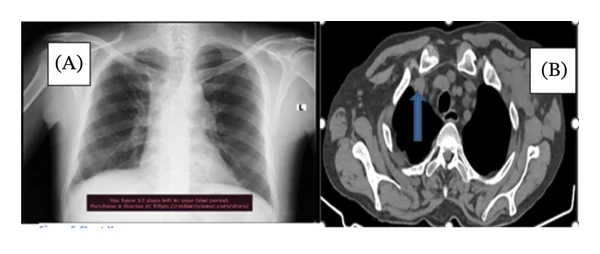
Chest imaging before and after immunosuppressive therapy: (A) a chest X‐ray with unremarkable findings before immunosuppressive therapy; (B) a noncontrast high‐resolution chest CT with multiplanar reconstruction on the mediastinal window, revealing mediastinal lymphadenopathy (blue arrows), highlighting radiologic evidence of pathology not apparent on plain radiography.

Chest imaging revealed unremarkable findings on the initial chest radiograph obtained before immunosuppressive therapy (Figure 4A). In contrast, the subsequent noncontrast high‐resolution chest CT with multiplanar reconstruction on the mediastinal window (Figure 4B) revealed multiple mediastinal lymphadenopathies (blue arrows), highlighting radiologic evidence of underlying pathology that was not apparent on plain radiography.

Diagnostic evaluation revealed nephrotic‐range proteinuria (19.7 g/24 h), severe hypoalbuminemia (0.47–0.7 g/dL), hyperlipidemia, and acute kidney injury with elevated creatinine and urea, consistent with nephrotic syndrome and renal dysfunction. Renal biopsy revealed diffuse mesangial expansion with Congo red positivity and green birefringence under polarized light, confirming the diagnosis of AA amyloidosis. Immunohistochemistry revealed strong serum amyloid A deposition, and immunofluorescence was negative for immunoglobulins, complement, and light chains. Excisional lymph node biopsy revealed necrotizing granulomatous inflammation highly suggestive of tuberculous lymphadenitis, and chest CT identified mediastinal lymphadenopathy despite an unremarkable chest radiograph. Together, these findings established a diagnosis of nephrotic syndrome secondary to renal AA amyloidosis due to EPTB.

## 6. Management and Patient Outcome

Immunosuppressive treatment was discontinued after the renal biopsy result arrived 10 days after initiation. Subsequently, the patient was initiated on standard anti‐TB therapy with rifampicin, isoniazid, pyrazinamide, and ethambutol (RHZE regimen) for 6 months, in line with national guidelines. Supportive care for nephrotic syndrome included furosemide for edema control and atorvastatin for hyperlipidemia treatment. Close monitoring of renal function was performed, and initial improvement was noted with stabilization of creatinine (1.8 mg/dL) and resolution of fatigue, flank pain, and constitutional symptoms.

**FIGURE 5:**
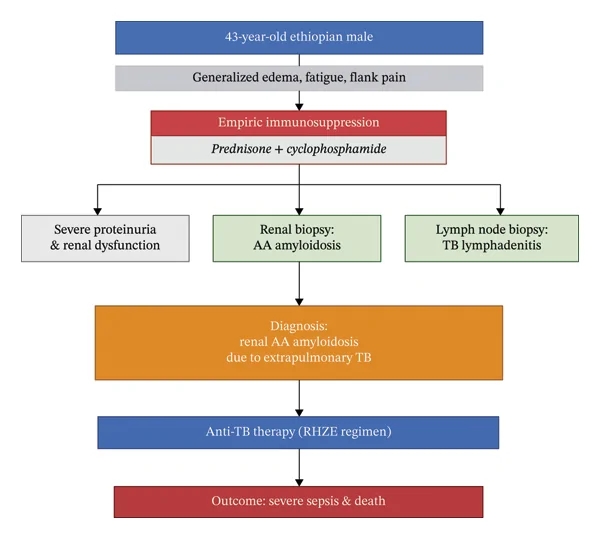
Clinical flowchart illustrating the diagnostic and therapeutic pathway of a 43‐year‐old Ethiopian male presenting with nephrotic syndrome secondary to renal AA amyloidosis due to extrapulmonary tuberculosis. The chart outlines the sequence from initial presentation and empiric immunosuppression to confirmatory renal and lymph node biopsies, initiation of anti‐TB therapy, and the outcome of sepsis and death.

Despite early clinical improvement, the patient returned three weeks later with severe sepsis of pulmonary origin, presenting with cough, chest pain, hypoxemia, leukocytosis (WBC 18,500/µL), and bilateral consolidation on chest imaging. Intensive management with broad‐spectrum antibiotics, including intravenous meropenem and vancomycin, vasopressor support, and mechanical ventilation, was initiated. However, the patient’s condition deteriorated and was complicated by septic shock and multiorgan dysfunction, and he died in the intensive care unit despite aggressive treatment (Figure 5). No postmortem evaluation was performed. However, prior exposure to immunosuppression may have contributed to the reactivation of latent TB and increased susceptibility to overwhelming infections.

In line with established guidelines, adult‐onset nephrotic syndrome requires histopathological confirmation before immunosuppressive therapy, as empirical treatment can be detrimental, as demonstrated in the present case [3]. Although AA amyloidosis may arise from various chronic inflammatory processes, our patient was evaluated for alternative causes, including autoimmune diseases, hepatitis B and C, and other systemic infections, none of which were identified. Renal biopsy confirmed AA amyloidosis, and lymph node histology demonstrated necrotizing granulomatous inflammation consistent with TB, strongly supporting TB as the underlying etiology. While biopsy remains essential in all cases of nephrotic syndrome, this report emphasizes that in TB‐endemic regions, clinicians must also maintain a high index of suspicion for TB and exclude infectious causes before initiating immunosuppression therapy.

## 7. Discussion

Nephrotic syndrome is a clinical entity characterized by edema, heavy proteinuria, hypoalbuminemia, and dyslipidemia, often leading to progressive renal dysfunction. Its etiology is diverse, ranging from primary glomerular diseases to systemic disorders and infectious causes, such as TB, syphilis, malaria, hepatitis B, and hepatitis C [3]. In TB‐endemic regions, secondary causes must be carefully considered before initiating immunosuppressive therapy, as overlooking infectious etiologies can worsen outcomes. The chronological sequence in this case is important to emphasize empirical immunosuppression with prednisone and cyclophosphamide was initiated before the renal biopsy results based on the presumptive diagnosis of high‐risk membranous nephropathy. Following the onset of lymphadenopathy and constitutional symptoms, further evaluation was pursued, and renal biopsy subsequently confirmed AA amyloidosis, while lymph node histology established TB lymphadenitis. Thus, immunosuppression preceded the histopathological confirmation. Since a postmortem examination was not performed, there are other possible causes for the death of this patient, such as disseminated TB itself and immune reconstitution inflammatory syndrome (IRIS). Prior immunosuppression may have contributed to the death, underscoring the diagnostic pitfalls and reinforcing the necessity of biopsy before therapy in adult‐onset nephrotic syndrome.

TB remains a major health burden in Ethiopia, with EPTB accounting for nearly one‐third of the cases [2]. Renal involvement is rare but clinically significant, often manifesting as sterile pyuria, hematuria, or secondary AA amyloidosis. Amyloidosis results from chronic inflammation, with serum amyloid A protein deposition in the renal tissue, leading to nephrotic syndrome and progressive renal failure [5, 6]. In developing countries, TB is the leading cause of renal AA amyloidosis, and delayed recognition contributes to a poor prognosis.

Our case highlights a critical diagnostic pitfall: empirical immunosuppression without histopathological confirmation of the diagnosis. The patient was initially treated with prednisone and cyclophosphamide for presumed high‐risk membranous nephropathy, which likely reactivated latent TB and led to lymphadenopathy and systemic symptoms. Similar cases have been reported in India, where patients with pulmonary TB later developed renal amyloidosis and nephrotic syndrome [7, 8]. Unlike these cases, our patient’s TB was initially unrecognized, underscoring the importance of thorough screening before immunosuppression in endemic regions.

The absence of routine TB screening contributed to the delayed diagnosis. Subtle symptoms, such as intermittent cough and pleuritic chest pain, were overlooked, and the chest X‐ray findings were unremarkable. Screening tools, such as the TST or IGRA, could have identified latent TB prior to immunosuppression, potentially altering the clinical course [3, 6]. In resource‐limited settings, where renal biopsy and advanced diagnostics are not always accessible, prioritizing TB screening is essential to avoid misdiagnosis and inappropriate therapy.

This case underscores the need for heightened clinical suspicion and context‐appropriate management strategies in TB‐endemic areas. Empirical immunosuppression without biopsy confirmation should be avoided because it risks reactivating latent infections and complicating patient outcome. Early identification of renal amyloidosis secondary to TB can improve the prognosis, while comprehensive diagnostic evaluation, including biopsy and TB screening, remains the cornerstone of safe and effective management [7, 9]. Furthermore, this fatal outcome illustrates how prior immunosuppression in a TB‐endemic setting can predispose patients to reactivation and overwhelming infection, reinforcing the need for biopsy confirmation and exclusion of infectious causes before the initiation of immunosuppressive therapy.

## 8. Limitations

This case report is limited by its single‐patient design, which restricts its generalizability to broader populations. The absence of postmortem evaluation prevented confirmation of the exact cause of death and the extent of systemic amyloid deposition in this case. Additionally, resource constraints limited access to advanced diagnostic tools, such as timely renal biopsy results, IGRAs, and TSTs, which could have aided earlier identification of latent TB.

## 9. Conclusion

TB‐associated renal amyloidosis is a rare but serious cause of nephrotic syndrome in endemic regions, and this case illustrates the dangers of empirical immunosuppression without renal histopathological confirmation. The patient’s initial misdiagnosis and premature use of prednisone and cyclophosphamide likely reactivated latent TB, delaying appropriate therapy and complicating the clinical course of the disease. In resource‐limited settings, clinicians must maintain a high index of suspicion for latent and EPTB, prioritize screening with TST or IGRA, and confirm the diagnosis with renal biopsy before initiating immunosuppression. Early recognition of TB‐related amyloidosis can improve outcomes, while avoiding unnecessary immunosuppression reduces the risk of reactivating latent infections and prevents fatal complications of the disease.

**Nomenclature**
AA amyloidosis: Amyloid A amyloidosis
AKI: Acute kidney injury
Cr: Creatinine
CT: Computed tomography
EPTB: Extrapulmonary tuberculosis
IGRA: Interferon‐gamma release assay
KDIGO: Kidney Disease: Improving Global Outcome
TB: Tuberculosis
TST: Tuberculin skin test
WHO: World Health Organization

## Author Contributions

Alemayehu Abebe Guji, Daniel Rebuma Bekele, Derebe Shashigo Leilago, and Getnet Amberber Degefu made contributions to the first draft’s conception, design, investigation, analysis, and writing. Alemayehu Abebe Guji and Birhanu Gudeta Geleta took part in the supervision, analysis, and edition of the data, as well as data curation. The draft was finalized by Alemayehu Abebe Guji and Kedir Negesso Tukeni.

## Funding

No funding was received for this research.

## Disclosure

The manuscript was modified and reviewed by all authors before being approved in its final form.

## Ethics Statement

Ethical approval was not needed; however, the confidentiality of participants’ information was kept secret and was used only for this publication without patients’ identification.

## Consent

Written informed consent was obtained from the patient’s next of kin for the publication of this case report and accompanying images. A copy of the written consent is available for review by the editor‐in‐chief of this journal.

## Conflicts of Interest

The authors declare no conflicts of interest.

## Data Availability

The data that support the findings of this study are available from the corresponding author upon reasonable request.

## References

[1] Bi K. , Zhao Y. , Lu S. , Xu K. , and Zhang Y. , Comments on the WHO Global Tuberculosis Report 2024: Progress, Challenges and Innovations for Achieving End TB Strategy by 2035, Infectious Microbes & Diseases. (2025) 7, no. 1, 1–4, 10.1097/im9.0000000000000169.

[2] Arega B. , Mersha A. , Minda A. et al., Epidemiology and the Diagnostic Challenge of Extra-Pulmonary Tuberculosis in a Teaching Hospital in Ethiopia, PLoS One [Internet]. (2020) 15, no. 12 December, 1–15, 10.1371/journal.pone.0243945. PMC7737896 33320897

[3] Rovin B. H. , Adler S. G. , Barratt J. et al., KDIGO 2021 Clinical Practice Guideline for the Management of Glomerular Diseases, Kidney International. (2021) 100, no. 4, S1–S276, 10.1016/j.kint.2021.05.021. 34556256

[4] Bansal B. , Sarkar A. , Barwad A. et al., Clinical Profile and Outcome of AA Amyloidosis-Associated Kidney Disease in India, Amyloid: The International Journal of Experimental and Clinical Investigation. (2025) 33, 1–10, 10.1080/13506129.2025.2573233. 41204056

[5] Karam S. , Haidous M. , Royal V. , and Leung N. , Renal AA Amyloidosis: Presentation, Diagnosis, and Current Therapeutic Options: A Review, Kidney International. (2023) 103, no. 3, 473–484, 10.1016/j.kint.2022.10.028. 36502873

[6] Hassen M. , Bates W. , and Moosa M. R. , Pattern of Renal Amyloidosis in South Africa, BMC Nephrology. (2019) 20, no. 1, 1–9, 10.1186/s12882-019-1601-x. PMC6842472 31706285

[7] Nassih H. , El Qadiry R. , Bourrahouat A. , and Ait Sab I. , Challenges in the Diagnosis and Management of Nephrotic Syndrome in a Child With Multisystemic Tuberculosis, Global Pediatrics Health. (2022) 9, 4–6, 10.1177/2333794x221078703. PMC8882937 35237712

[8] Balwani M. R. , Kute V. B. , Shah P. R. , Wakhare P. , and Trivedi H. L. , Secondary Renal Amyloidosis in a Patient With Pulmonary Tuberculosis and Common Variable Immunodeficiency, Journal of Nephropharmacology. (2015) 4, no. 2, 69–71. PMC5297488 28197481

[9] Dahyana C. A. , Gustavo Z. V. , Arbey A. A. et al., Renal AA Amyloidosis Secondary to Disseminated Tuberculosis: A Case Report and Literature Review, Journal of Clinical Pathology. (2025) 13, 1–5, 10.52338/tjocp.2025.5121.

